# Progress in Neurology

**Published:** 1902-06-28

**Authors:** 


### PROGRESS IN NEUROLOGY.

(*Continued from page* 206.)

**Epilepsy.**—In the course of a lecture on epilepsy Broadbent[3] says that a low tension pulse is characteristic of the disease; it is a part of the general sluggishness of the organic operations, like the pallor which is common and the dull complexion and flabby skin. A pulse of unduly high tension, for the age of the patient, is a ground of hope of recovery. As regards treatment by drugs, when the intervals between the fits are six weeks and over, bromides should not be given regularly and continuously unless there is some other indication for their employment, such as sleeplessness or excitement. If the attacks recur punctually at definite intervals, bromides may be given when they are due, or again if recognised premonitory symptoms occur, or if some special incident, which by experience the patient knows will probably bring on an attack, is going to happen. When the fits are frequent, and especially when it is clear that bromides exercise a restraining influence, they must be given in such amount as may be found necessary and at such hours as may be suggested by the time at which the fits are liable to occur. Thus, if the fits come on in the night or early morning a dose should be given at bedtime.

**Mental Changes in Visceral Disease.**—In the Goulstonian Lectures for 1901 Head[4] discusses a group of changes in consciousness associated with the reflected pain of visceral disease, and with accompanying tenderness of the skin in definite areas. The mental changes described are the following. A *sense of ill-being*, which occurs paroxysmally without obvious cause and drives the patient from companionship in search of solitude, or to huddle under the bed clothes; weeping or desiring to weep, and often dumb to questioning; haunted by vague idea of impending evil, and in some cases visualising the distant home as dull, dirty, disordered, and cheerless. The attacks may be short, the ebb and flow of frequently recurring seizures may last several days, or some of the states may recur at longer intervals. A *mood of exaltation*, sudden feelings of being able to do, and then attempts to do, actions far beyond the physical state. *Suspicion* that their nearest relatives and friends are speaking ill of them—saying, for instance, that they are lazy, idle, worthless, or shamming. *Hallucinations*, (*a*) visual, chiefly imperfect, indefinite draped figures of a white, black, or grey colour—never of the colours of life, as in the insane; (*b*) auditory, which are never of articulate voices; (*c*) smell. These mental changes are not accounted for by fever, loss of weight, and vascular disturbance, or anæmia. The only factor universally present is reflected visceral pain, with its superficial tenderness, and its associated visceral headache. The mental states described above have been classed as hysterical, but Head now seeks to relate them to their proper position.

**Volkmann's Contracture.**—Dudgeon[5] has described four new cases of Volkmann's contracture, and collected all the previously recorded instances of this rare condition. The disease may be defined as a contraction of the fingers and sometimes of the wrist, which comes on rapidly, with loss of power which is not absolute in the forearm muscles, after a severe injury, usually in the region of the elbow-joint, generally in young children. The deformity is due to changes in the flexor muscles without injury to the peripheral nerves, caused in many cases by tight bandaging and the pressure of splints—it is an ischæmic paralysis. Pressure sores have occurred in about half the cases; their absence in the other half shows that they are not the cause of the condition, but simply evidence of the pressure to which the parts have been subjected by the splints. The condition is probably due to a combination of large effusion into the soft parts and splint pressure. The symptoms come on rapidly—in half a day, or less in severe cases, there is a rapid onset of paralysis with contracture. There is rarely any pain. The resultant deformity is very characteristic. With the wrist extended, the metacarpo-phalangeal joints are extended also; the interphalangeal finger joints and the terminal joint of the thumb are, however, strongly flexed, so that the tips of the fingers touch the palm, and no reasonable amount of force straightens them; but as soon as the wrist is flexed the interphalangeal joints can be easily extended. In very bad cases the wrist is strongly flexed and incapable of extension. The flexor muscles of the forearm are firm, hard, and much wasted. Sensation may be normal, or there may be anæsthesia—this is dependent on the condition of the peripheral nerves. There are no true trophic lesions, but the hand is frequently cold and blue, and may swell when the arm hangs down. The joints do not become anchylosed; in well-marked cases the bones of the forearm do not develop, just as in anterior poliomyelitis. A most characteristic feature of the condition is that the electrical reactions of the muscles and nerves are normal. Volkmann considered that the condition is due to diminution of the blood supply, *i.e.* is ischæmic in origin, and its degree is dependent on the length of time the circulation of blood has been arrested. The muscle fibres perish in a greater or less degree owing to deprivation of their oxygen. The rigidity and contracture are made worse by the subsequent production of fibrous tissue in the muscles. The diagnosis from paralysis of the ulnar, median, or musculo-spiral, nerves is made by (1) the characteristic history, (2) the onset of paralysis and contracture simultaneously, (3) the normal electrical reactions, and generally (4) the absence of any loss of sensation. The prognosis is doubtful; whilst Volkmann said it was hopeless, all Dudgeon's cases completely recovered. The treatment is by long continued massage, with, if necessary, lengthening of the flexor tendons or shortening of the forearm bones by operation.

[3] Brit. Med. Jour., Jan. 4, 1902. [4] Brit. Med. Jour., Jan. 18, 1902.
[5] Lancet, Jan. 11, 1902.

